# Feasibility and Acceptability of Computerised Cognitive Training of Everyday Cognition in Parkinson's Disease

**DOI:** 10.1155/2019/5258493

**Published:** 2019-07-22

**Authors:** S. J. Smith, I. McMillan, I. Leroi, C. L. Champ, S. Barr, K. R. McDonald, J. P. R. Dick, E. Poliakoff

**Affiliations:** ^1^Centre for Dementia Research, Leeds Beckett University, Leeds, UK; ^2^Division of Neuroscience and Experimental Psychology, School of Biological Sciences, University of Manchester, Manchester, UK; ^3^Greater Manchester Mental Health Foundation Trust, Prestwich, UK; ^4^Global Brain Health Institute, Trinity College Dublin, Dublin 2, Ireland; ^5^Greater Manchester Neurosciences Centre, Salford Royal NHS Foundation Trust, Salford, UK

## Abstract

**Objectives:**

We piloted a computerised cognitive training battery in a group of participants with Parkinson's disease without dementia to investigate the relevance of the training to daily life and the feasibility and the acceptability of the tasks. Previous studies of CT have had limited success in the benefits of training, extending to improvements in everyday function. By taking a pragmatic approach and targeting training to the cognitive skills affected by Parkinson's disease (planning, attention, and recollection), whilst using tasks that emulated real-life scenarios, we sought to understand whether participants perceived the training to be effective and to identify the elements of the training that elicited beneficial effects.

**Methods:**

Four participants completed a cognitive training session comprising three distinct tasks 5 days a week over two weeks. Participants completed baseline questionnaires examining health-related quality of life, everyday cognition, and apathy before the training period, after the last session, and two weeks after the last session. An interview was held after participants had completed the training.

**Results:**

The findings indicated that participants felt the training was acceptable, enhanced their awareness, and encouraged them to monitor their thinking abilities. The group interview indicated that the training was feasible; participants felt the tasks had potential to improve everyday performance, but more supporting information should be provided to facilitate this transfer. Responses to the questionnaires reflected these findings, indicating improvement for some participants' cognition and quality of life. Objective measures supported the subjective reports; there were improvements in some but not all domains. Performance on the planning and recollection tasks improved over the training period, and the evidence for improvement on the attention task was mixed.

**Conclusion:**

This study has found that pragmatic computer-based training with real-life outcomes is both feasible and acceptable and should be evaluated more extensively using controlled methods.

## 1. Introduction

Although Parkinson's disease (PD) is primarily a motor disorder, cognitive impairment is also common early in the disease and in individuals who do not meet the criteria for dementia in PD [1]. Cognitive decline has been shown to contribute significantly to quality of life [2, 3]. It has also been demonstrated that reduced activities of daily living are associated with decline of cognitive function in PD [4]. Neuropsychological tests have indicated impaired attention and executive, visuospatial, and memory function [5] and strategy use [6, 7] in PD. These cognitive skills are implicated in activities of daily living such as working, shopping, planning appointments, medication adherence, and social interactions, and people with PD report making more everyday cognitive errors [8]. Such deficits may lead to reduced quality of life [9] and social isolation [10].

Current treatments for cognition in PD are mostly pharmacological. However, treatments, such as the use of rivastigmine, the only cholinesterase inhibitor specifically licenced in the UK for use in PD, have been shown to only have moderate effects [11]. Taken with the complex pharmacological regimen associated with the management of Parkinson's disease and the growing body of evidence for nonpharmacological approaches towards the management of cognitive problems in other clinical groups, there is a compelling argument for nonpharmacological cognition-enhancing therapies in PD.

This study aimed to investigate the feasibility and acceptability of a two-week computerised cognitive training (CT) programme for people with PD. Findings regarding the outcomes of cognitive training programmes for people with PD have so far been promising; however, there is a great deal of variability regarding the approaches to training and the ways in which the outcomes have been measured [12]. In a recent review, Glizer and MacDonald [12] identified 13 studies of cognitive training in PD conducted between 2000 and 2014. The reviewed findings suggest that cognitive training can elicit short-term, moderate improvements in cognitive functions. However, the variability of the approaches makes it difficult to identify the mechanisms of improvement and degree of impact.

The review [12] included paper-based and computerised tasks. The computerised cognitive tasks were varied in the cognitive domains, targeting and eliciting a range of improvements. For example, computerised cognitive training that targeted attention, abstract reasoning, and visuospatial skills elicited improvements in verbal memory and abstract reasoning that were maintained at 6 months [13]. Similarly, Mohlman et al. [14] found that computerised attention tasks (90 min a week) elicited benefits for executive function and verbal memory. The aforementioned studies relied on standardised neuropsychological outcome measures to demonstrate the benefit. The issue with standardised neuropsychological outcomes is that they do not relate to functional benefits, and therefore, the feasibility of cognitive training as a mechanism for improving everyday function is not established. Edwards et al. [15] delivered computerised speed of processing training and observed improvements in field of view ability at 3 months, but no corresponding improvement in self-reported cognitive function using a cognitive self-report questionnaire. París et al. [16] used smart brain training across a range of cognitive domains and found the CT group improved on several cognitive outcomes compared to a control group, who received speech and language therapy; however, no benefits were reported for self-completed questionnaire reported mood or quality of life.

In terms of cognitive function, the findings of these studies suggest that positive outcomes in cognition can be achieved by providing training that targets the specific cognitive functions affected by PD. This suggests the existing “off-the-shelf” approach to cognitive-enhancing interventions, such as those that target Alzheimer's disease, would not be suited to a population with PD [17]. The study presented in this manuscript utilised everyday cognition tasks that target functions known to be affected by early PD-related cognitive impairments (attention, recollection, and planning). This includes abilities that have previously been successfully targeted [12]. A further novelty of the study was that the tasks were designed to emulate real-life scenarios, an approach endorsed by previous studies of cognitive training (e.g., Grewe et al. [18]) in an attempt to enhance the generalisability of the training.

A key interest of the current pilot study was exploring the personal experiences of the participants in relation to the training to establish the feasibility and acceptability of the training. The aforementioned studies have demonstrated that a limitation of a cognitive training approach is the lack of generalisability to real-world functions [19], and the small impact on meaningful personal outcomes, such as self-reported function and mood. Despite this, the reported experience of participants' “use” of cognitive training in the real world has not been investigated, which may provide clues as to why the training is/is not impacting real-world function. There is some evidence that strategy use may play a role in these transfer effects: Ceresa et al. [20] attempted to establish the contribution of strategy use to transfer effects by delivering three conditions of cognitive training: CT alone, CT and transfer training (strategies), and CT and transfer training (strategies) and motor skill training. The multimodal training elicited more pronounced effects and increased quality of life.

The current study explores transfer effects to everyday function by using an outcome measure designed to be sensitive to changes in everyday function in PD (PD-Everyday Cognition Questionnaire) [21]. This measure has not previously been used in studies of CT in PD. In summary, this pilot has taken a pragmatic approach to measuring the outcomes of the training tasks in order to attempt to identify transfer effects of the training to everyday function and investigate the potential mechanism of change by qualitative investigation of the outcomes of the training and strategy use, as well as the acceptability and feasibility of the training. We focused on individuals at an early stage of disease progression, without a diagnosis of dementia in PD (PDD) or cognitive impairment that would meet the criteria for a diagnosis of PD-MCI.

## 2. Methods

### 2.1. Participants

Six participants were initially recruited for the study. One participant completed test versions of the tasks which were subsequently refined. One participant withdrew before commencing the training, due to an unrelated health condition. The data presented are of the four participants (female = 1) who completed the refined cognitive training tasks.  Table 1 lists participant demographics. The age of the participants ranged from 56 to 74. All had a diagnosis of idiopathic PD (Hoehn–Yahr score ≤ 3) [22], experiencing mild to moderate motor symptoms. None of the participants had motor symptoms of sufficient severity to interfere with completion of the tasks. Exclusion criteria included the following: the presence of significant auditory or visual impairments; marked cognitive impairment indicated by scores less than 82/100 on the Addenbrooke's Cognitive Examination-Revised scale (ACE-R [23]); meeting clinical criteria for PDD [9]; a significant level of depression indicated by a score greater than 11/30 on the Geriatric Depression Scale (GDS [24]); neurological diseases (other than PD); significant psychiatric illness warranting inpatient treatment; or a history of serious head injury. Premorbid IQ and verbal ability were measured using the National Adult Reading Test [25] and Mill Hill verbal fluency test [26], respectively. The performance of the participants on the ACE-R scale does not suggest the presence of MCI based on the proposed cutoff score of 85.5 [27, 28]. All participants were receiving combined levodopa and dopamine agonist therapy and had been stable on their medication regimen for at least four weeks prior to the start of the training. One participant was also prescribed monoamine oxidase inhibitor B (selegiline), and three participants used an NMDA antagonist (amantadine). The study was approved by the local NHS Research Ethics Committee (11/NW/0420), and all participants had the capacity to consent.

### 2.2. Procedure

The procedure comprised three parts over a period of six weeks. Prior to commencing, participants completed a screening visit to ascertain their suitability to take part in the study. Baseline measures were taken before the cognitive training. The training consisted of 10 sessions over a period of two weeks. Follow-up sessions were completed at one and three weeks after training, following which a group interview was held. Participants were given the opportunity to complete the training programme at the University of Manchester but, excluding one session, elected to complete all training at home. Participant 3 was only able to complete 8 of the training sessions due to personal commitments.

#### 2.2.1. Baseline

The baseline visit was conducted within 6 weeks of the screening visit. The baseline visit included the PD-Everyday Cognition Questionnaire to assess self- and carer-reported problems in everyday cognitive functioning in PD in memory, attention, and executive function [23]; the Parkinson's Disease Quality of Life Inventory-39 [29], which comprises 39 questions covering 8 aspects of quality of life; and the Lille Apathy Rating Scale [30].

#### 2.2.2. Cognitive Training

Visits two to eleven were completed over a period of two consecutive weeks (excluding weekends) following the baseline visit. At each visit, the participants completed 1-2 hours of cognitive training on a laptop with a 15″ touch screen. The researcher was always present during the training. Participants responded to stimuli presented on the laptop screen by tapping the screen or responding on a keyboard. The cognitive training comprised three distinct tasks: attention, planning, and recollection training. The researcher set up the tasks on the laptop and was present during completion to answer any questions.

*(1) Attention Training*. This was delivered in three sections, which pertained to central attention, divided attention, and inhibiting attention. The tasks used pictures of cars and road signs as stimuli. For central attention, participants were first presented with a central stimulus (one of two different cars) in the centre of the screen. After a 1 s masking stimulus, participants were shown one of the two cars and asked to decide which car they had previously seen. To measure divided attention, the task was repeated, but at the same time as the central stimulus (car) is presented, a second stimulus is presented in the peripheral vision at one of 8 locations (a star). The participants had to identify both the central stimulus and the location of the peripheral stimulus. In the third cognition (inhibiting attention), a peripheral stimulus (a star) was first briefly presented. Participants must inhibit orientating to this location (cf. Deijen et al. [31]) and instead identify a second target (road sign) presented 200 ms later in the opposite location when given a choice of stimuli that has been presented. The difficulty of these tasks increased according to the participant's performance, based on the PEST algorithm [32], which honed in on the level at which participants could perform 75% correct. The difficulty level was manipulated via the presentation time of the target stimulus: the easiest time being 16 ms and the most difficult 1930 ms. The first two tasks began at 965 ms, and the last task began at 1335 ms. The performance measures for these tasks were the threshold presentation times that the participant reached.

**Figure 1:**
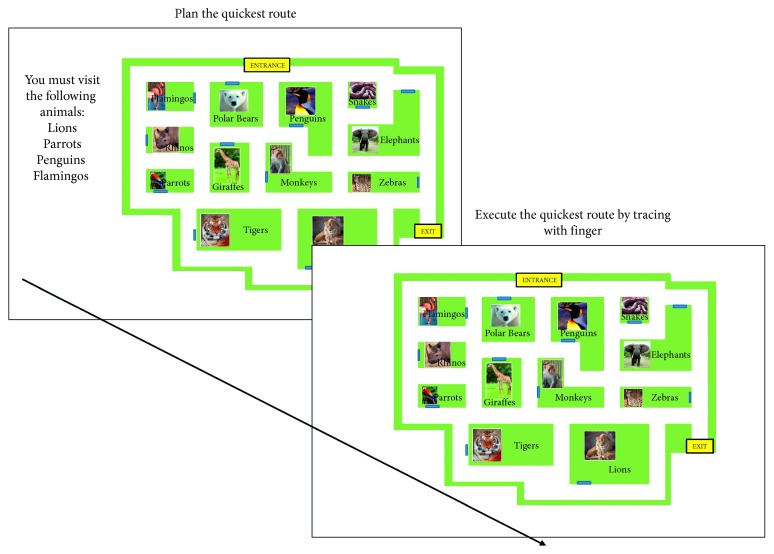
Planning task.

*(2) Planning Training.* This involved planning and executing routes through three environments: a zoo, a museum, and a supermarket. Participants first viewed a map of the environment along with a list of places/items that they needed to visit/pick up within the scenario. With the map on screen, participants were asked to organise the list according to the order in which they would visit the places/pick up the items to give the quickest route through the environment. Once they had planned the route, participants were shown the map again along with the places/items on the list and asked to execute their chosen path by clicking on points within the environment (see Figure 1). As they touched the route, each segment of the path changed colour. The task difficulty increased after each completed route by giving participants longer lists of places/items. The task began with two places/items (Level 1), and the maximum of places/items was 7 (Level 7). Participants had to complete two routes at the same level before progressing to the next level. In each session, participants had 15 minutes to complete as many routes as possible on the same map (the map and environment changed each day). The performance measure was the difficulty level reached at the end of the 15-minute period.

**Figure 2:**
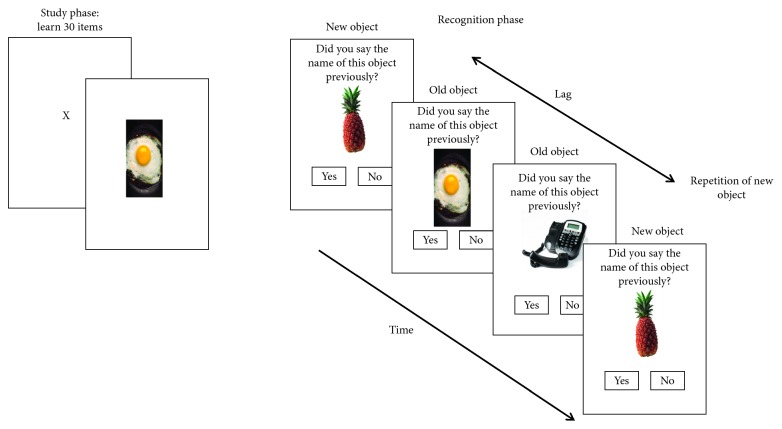
Recollection task.

*(3) Recollection Training.* This was based on the incremental difficulty approach to training recollection [33]. With a study phase and a recognition phase, participants saw 30 pictures of everyday objects (e.g., a balloon). Each object was presented on-screen for 3 seconds, following a 1-second fixation cross. In the recognition phase, participants were presented with the 30 objects shown on the study list (“old” objects) along with 30 distracter objects (“new” objects). The objects were presented one at a time, and participants had to decide whether they were “old” (had been on the study list) or “new” (see Figure 2). They were specifically instructed that they should only press “old” if they had previously studied the item. If they had responded correctly, they saw a green tick for 1.5 seconds; otherwise, a blank screen was presented for the same duration. The distracter objects were each presented twice, requiring participants to distinguish between “old” items and “new” items that had been presented more than once. The task difficulty was manipulated by the delay that occurred between the repeated presentation of the new distracter items. There were 10 difficulty levels in total. For example, at level 1, there was a lag of one, two, or three such that one, two, or three objects were presented before the “new” object was repeated. Participants completed the task once per visit. If they made fewer than two errors, they passed the level and progressed up two levels at the following visit. If they made more than two errors, they moved down a level at the next visit (unless on level 1). If participants passed a level they had previously failed, they progressed up one level at the next visit. The performance measure was the level reached at each visit.

#### 2.2.3. Follow-Up

The questionnaires administered at baseline were repeated at both follow-up visits, along with a questionnaire regarding the participants' perceptions of the training and their subjective experience of any cognitive and functional improvement. The questionnaire regarding the participants' subjective experience of the training comprised the following questions: (1) What did you think of the trainings tasks? (2) Can you think of any changes that you would like? (3) Do you have your own computer? If yes, would you have liked to download the training exercises on your own computer? If no, would you have been interested to learn to run the training exercises on a computer so you could choose when to do them yourself? (4) Have there been any changes in your daily activities which have come about as a result of the training?

*(1) Interview.* An interview with two participants was conducted after the training to capture subjective experiences, including perception of any changes in cognitive function and functional outcomes. Two participants were unable to take part due to illness. The interview schedule was developed to include questions regarding cognitive and functional changes (feasibility), as well as the acceptability of the programme. The schedule included questions concerning how the participants felt about the tasks, effects on thinking abilities, effect on the ability to carry out their daily tasks, and perceived importance of the tasks. The interviews were conducted by the staff trained in qualitative approaches, recorded, and transcribed for the purpose of analysis.

## 3. Results

This section is presented in three parts: (i) an exploratory comparison of self-reported measures before and after training; (ii) the interview findings; and (iii) an overview of participants learning on each of the training tasks by mapping the trajectory of the main outcome measures on each of the tasks over the training period.

### 3.1. Self-Reported Measures before and after Training

The findings from the self- and carer-reported measures are presented in Table 2. All measures were taken at baseline (Time 1), immediately after the final session (Time 2), and two weeks after the final session (Time 3). On the PD-Everyday Cognition Questionnaire, self-reported performance on the subdomains attention, memory, executive, and strategies was examined at T1, T2, and T3. No formal statistical analysis was undertaken due to limitations of sample size. However, all participants reported more attentional errors at T3 compared to T1 on the attention subdomain. Conversely, three participants reported fewer errors at T3 compared to T1 on the memory and executive subdomains. The strategy use subdomain seemed to remain relatively stable; two participants reported more strategy use at T3 compared to T1, of which one was considerable. The overall carer scores indicated that three of the four carers reported fewer errors on behalf of their partners at T3 compared to T1.

Overall, there was no apparent trend across participants in terms of improvements before and after training on the Parkinson's Disease Quality of Life Inventory-39 [27]. The Lille Apathy Rating Scale [30] scores overall indicated a trend towards participants becoming less apathetic, and 3/4 of participants had a lower score at T3 compared to T1 (higher scores represent a greater degree of apathy).

In addition to the standardised questionnaires, participants also were able to give open comments which are reported in Table 3.

### 3.2. Interview

Two of the participants who completed the training took part in the group interview, along with one participant's spouse. The interview was led by a researcher who was not involved in data collection and was unknown to the participants, although a researcher known to the participants was present. Data analysis from the interview was conducted according to basic thematic analysis principles [34]. Although the interview schedule directed the participants to address specific issues, the analysis used in vivo coding to ensure that participants' own words were used to capture their responses. After transcribing the interview, thematic analysis was conducted that generated a list of codes and definitions (see Table 4). The codes were then collapsed into four major themes, as presented below.

#### 3.2.1. Usefulness

For the purpose of the interview, cognition was referred to as thinking abilities. Participants noted repeatedly that they considered the issue of “thinking” (the topic of the training) was important to them. One participant noted that this was important for people with PD “cos it's not just the physical side, it messes about with your brain sometimes doesn't it.” Another participant felt that this was “more than the physical, with the problems that I have had.” In general, the participants enjoyed doing the tasks: “I enjoyed doing them, it kind of gives you a purpose each day,” “[it] makes you feel like you are doing something positive.” There was consensus between the participants that they felt the training had elicited benefits in terms of performance on the tasks; however, a recurrent theme in the discussion was that they found it difficult to transfer the task-based improvements to real life. For example, “I certainly got better at some of the exercises without a doubt, and not just based on remembering the exercise, rote memory, but actually the concept of the exercise, and what was intended by it and ways of going at it. Particularly the route planning one at the end, but I couldt say that I could extrapolate that then to anything.”

An interesting issue raised at several intervals was usefulness of the tasks as a method of monitoring performance and providing useful feedback that could be used outside of a training paradigm.

Mr A: but it would be quite useful to have them as a monitoring, sort of dip test, because you could practice these skills in other ways as well, but having something that captures whether you are doing as well as a month ago or less well than a month ago, which might then make you do the exercises more often would be quite welcome.

Mrs L: I think that is a good point actually because you can't very easily measure whether you are getting better or worse. I have been doing it by writing a sentence, the same sentence to see how long it takes to do it, but something like that would help you to measure it as well.

#### 3.2.2. Informational Issues

The participants repeatedly came back to the issue of more information being provided both in relation to the purpose of the tasks and about the thinking abilities that the tasks represent. One comment alluding to this was as follows: “[participant would like] something that said, almost like a job spec: this is what you are going to do, and this is what you can look to achieving, or this is what you can expect to improve or, you know this is the outcome.” Further to this, the participants felt that it would be useful to know how they could “apply that now to making my memory better,” such that if they were given information about the tasks, it would be clear how the computerised tasks related to real-life scenarios.

The informational issues related closely to the theme of strategy use, with one aspect being the request for more feedback on the performance on the individual tasks; online feedback was built into the memory task but not the others.

#### 3.2.3. Strategy Use

It was very clear that both participants had developed internal strategies to help them perform the tasks, “the memory thing, you know the list and the pictures, if I spoke out loud about what was on the pictures, what I was seeing, it helped me to remember them. So when I did you know the words, in the introductory test, then pictures and then words again. And I found that if I said something about the words then, and I learnt that from the test.” The participants were also aware that the feedback would be useful in helping them to develop their own strategies:

Mr A: the easier example is the one that erm, speaking out loud whether or not you have seen something in the list earlier, and it says yes or no, and it says you have got 80 of them all or whatever the number was, just that, because you can then start logging it and see ….

Mrs L: actually maybe that's why I liked that one because you got an immediate result, yes.

They would like to see more of this feedback (as outlined above). In addition, they felt that more information in general about the purpose and aims of the task would be useful to develop strategies. For example, “If you understand the intention and how the results are to be measured, you may have enough to form your own strategy. If you're going in blind, you have got to do it a few times as [Mr A] said earlier, what's going on in order to conceptualise and deal with it.”

Whilst they felt that information about strategies that might be useful would be helpful, they were clear that they did not wish to be told which strategies to use “if it is there is an option for people to use or not, because everybody works in different ways don't they? Or in a few different ways because people have different needs in terms of that knowledge support at the beginning, and will work out their own strategy anyway if that is what they need to do.”

#### 3.2.4. Practical Issues

As outlined below, there were some issues with regard to the application of the technology, which were identified and discussed by the participants. Participants felt that it would be of benefit to have more optionality in terms of the interface that the tasks used and the types of technologies available. This was felt to be a particularly important consideration for people with PD who may benefit from using some types of technologies over others.

Mr A: I use the Ipad, so it could go on there; I think that in my head would lend itself the best, to that range of things better than a PC, but a PC would do it as well with the button pushing facilities.

Fac: but if you had a choice between doing something on a PC and having a touchscreen, do you think the touchscreen would be easier?

Mrs L: Yes

Mr A: Yes it would for me at the right time of day, and at another time of the day it wouldn't, and a lot of people with Parkinson's … some people may not be able to use the tracking, but most people have times of the day where they could manage.

### 3.3. Training Measures

#### 3.3.1. Attention Training

**Figure 3:**
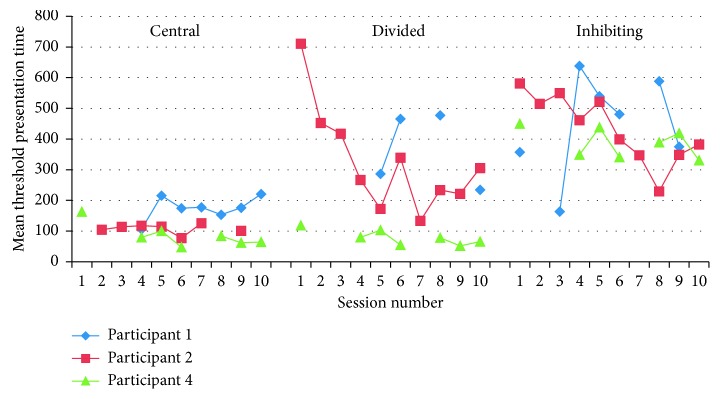
Threshold presentation times on the three attentional training subtasks.

The mean threshold presentation time is presented separately for each subtask (see Figure 3). There were some problems in recording the data for some participants. For several sessions, a problem with the touch screen meant that participants' responses were not recorded properly, so they were unable to complete the task. On a small number of occasions, the algorithm overestimated their threshold; if the participant was tired or failed to concentrate for a period of time, they could appear to be performing at threshold (75%) longer stimulus duration than they actually required. Sessions where there were technical problems were excluded from the analysis, and in an attempt to exclude overestimates, values > two standard deviations from the remainder of the participant's responses are excluded. Participant 3 has not been included in this analysis, as after the training, this participant reported difficulties seeing the stimuli; thus, we cannot be confident that they were able to complete the task.

There were insufficient data to conduct formal statistics, but overall there appeared to be no improvements on the central attention tasks. However, for the divided attention task and inhibiting task, the threshold presentation time appears to decrease over the sessions, signifying improvement in performance.

#### 3.3.2. Planning Training

**Figure 4:**
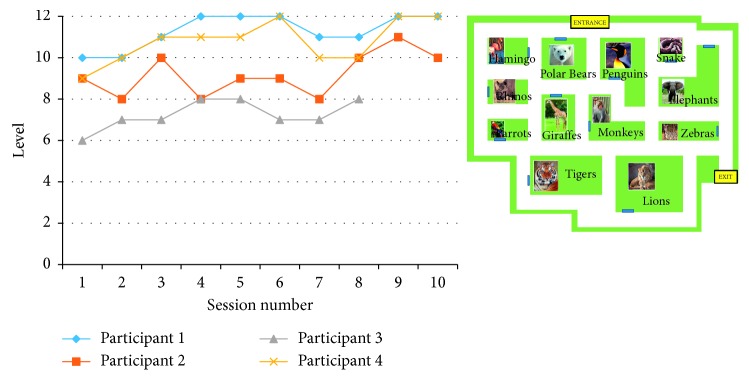
Level reached by participants in the planning task. Inset shows an example map of a zoo.

The difficulty level reached in each session increased for all participants from session 1 to session 10: this indicated that all participants improved from the first to the last session Figure 4. However, the task did appear to be subject to ceiling effects, with participant 1 performing at the highest possible level in session 4.

#### 3.3.3. Recollection Training

**Figure 5:**
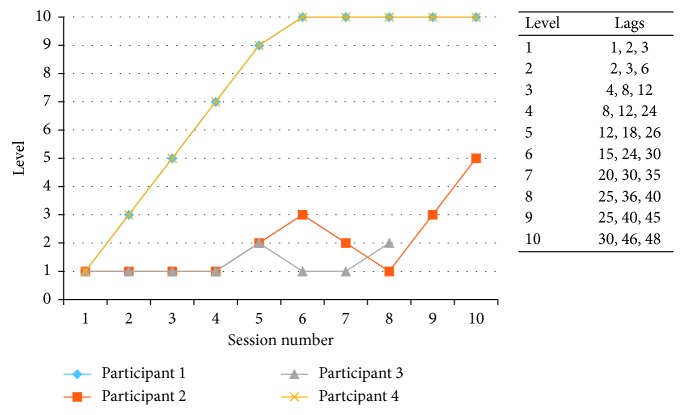
Level reached by participants in the recollection task.

The maximum level that participants reached on the recollection training task is presented in Figure 5. There was a broad range of performance on this task with participant 3, in particular, struggling. There was also an issue with participants 1 and 4 performing at ceiling, indicating that task difficulty was an issue. However, all participants demonstrated a learning curve across the sessions.

## 4. Discussion

In this exploratory study, through examining participants' subjective experience of the training, we were able to determine that a pragmatic computerised approach to cognitive training is both feasible and acceptable. The qualitative data from the questionnaires and focus groups indicated that the participants felt the training had a positive impact on their thinking abilities and that there were some transferable effects of the training to their everyday life. The participants also made several suggestions for improvements that could be made to the training, which points towards the acceptance of the general approach.

The open comments provided by one participant suggested that there were clear transferable effects of the training to [his] everyday functioning, specifically the implementation of projects that involve planning, such as going on holiday. However, the findings from the group interview suggested that although they felt that their thinking did improve over the training period and that they were more aware of their thinking abilities, the training did not have a significant impact on/transfer to everyday life. Overall, there was some tentative feeling that the transfer of improvements on the tasks to everyday functioning would be improved by (1) having a longer period of training and (2) having more information about the purpose of the tasks and why they should be helpful.

This second point relates back to the utility of the training as a kind of monitoring tool for participants. There was a feeling that providing more information about cognition can enhance participants' awareness of cognition and thus their ability to monitor their performance. The issue of information provision was central to this function for the participants, such that they felt this information would enhance their ability to make the most of their thinking abilities. It should be noted that the educational material alone is unlikely to improve cognitive performance, but it may provide a useful adjunct. This can be seen in a study of MCI (without Parkinson's disease), where memory improvements were observed when educational information (regarding memory and aging) was provided alongside memory training, but improvements were not produced by providing educational information alone [35].

Providing further supportive information about a task such as above may help people develop their own strategies. Similarly, the structure of a task itself might aid the development of strategies; in the planning task, participants were asked to structure the list according to the quickest route before they engaged in the task. This is particularly pertinent as studies have previously indicated that people with PD report less strategy use than age-matched controls [9]. The participants in the current study did not want to be provided with specific strategies and reported coming up with their own strategies in the recollection subtask. The participants also felt that they might be better able to come up with strategies given more information about the tasks, fitting with the notion of taking an individualised approach [36]. Previous research supports this assumption and has found that as long as sufficient time is provided for solving the task, patients with PD do not show a general deficit in the ability to internally generate a strategy, although this does depend upon cognitive load [37]. Interestingly, the questionnaire findings in this study suggest that strategy use increased for half of the participants and increased significantly for one participant.

The exploratory analysis of the quantitative data showed modest improvements on performance on some of the cognitive training tasks over the sessions. There appeared to be steady improvements on the recollection training task, similar to those previously reported in older adults [29], and in the planning task. These were positive findings, which support some of the earlier literature that suggests taking an individualised approach to cognitive training in a population with a specific profile of cognitive impairments is important [36]. However, both the recollection and planning tasks were subject to ceiling effects for some participants.

In contrast to the quantitative findings, for some of the participants, the self-reported questionnaire scores suggested that they experienced more cognitive errors after the training had finished. For example, scores on the attention subdomain (PDEQ) were higher at T3 compared to T1. Similarly, two of the four participants had a higher score on the PDQ-39 at T3 compared to T1. One interpretation of this finding is that giving participants the questionnaires at T1 (before the start of the training) had the effect of enhancing awareness of cognitive problems. This was mirrored in the open comments given at the end of the questionnaire, indicating that one participant and their partner felt that completing the questionnaire in itself made them more aware of [his] behaviour and memory. Similarly, in the focus group, one participant suggested that the training in itself made them more aware of their thinking abilities and deficits. In this case, a lower or higher score on questionnaire measures may be reflective of improvements in awareness, an overall positive outcome, since people who have greater awareness of the memory are more likely to benefit from cognitive rehabilitation [38]. It has previously been found that people with PD who have greater awareness of prospective memory perform better on prospective memory tasks [39]. This could be further investigated by using metacognitive measures such as the Memory Awareness Ratings Scale (MARS [40]) at the outset to estimate the accuracy of people's metacognition before and after the training. Indeed, one participant felt that the tasks would be useful as a monitoring tool, such that enhancing awareness of thinking abilities would act as an impetus to engage in more cognitive activities or to use strategies.

There were several limitations of this study including small participant numbers and difficulties regarding application of the technology. Nonetheless, the findings demonstrated that participants found the approach to be acceptable and led to general improvements in their thinking abilities. Some participants reported that there was limited transferability from the cognitive training tasks to their real-world function, although there was consensus regarding the potential for such effects. This would depend on being provided with more explicit information about the tasks to help inform the development of their own cognitive strategies. Incorporating such information might be a consideration in developing future cognitive training paradigms. The findings also strongly related to the issue of awareness and monitoring; the participants themselves felt that the training tasks could be useful as a mechanism for monitoring their own thinking abilities and enhancing their awareness. This in turn would ensure that they are better equipped to benefit from cognitive rehabilitation programmes. In summary, the findings support the notion that complementary cognitive enhancing therapies are a feasible and acceptable alternative/adjunct to pharmacological interventions.

## Figures and Tables

**Table 1:** Participant demographics.

	Participant 1	Participant 2	Participant 3	Participant 4
Age	56	58	74	67
Gender	Male	Male	Male	Female
Years of education	13	14	16	16
Addenbrooke's Revised Version A (0–100)				
Attention/orientation (0–18)	18	18	18	18
Memory (0–26)	23	19	18	21
Fluency (0–14)	14	11	11	14
Language (0–26)	26	24	26	26
Visuospatial (0–16)	14	14	15	16
Total	95	86	88	95
NART				
Errors	9	20	8	2
Estimated IQ	118	111	118	126
Mill Hill vocabulary test	27	13	22	25
GDS	4	4	4	1
Disease duration (years)	16	8	12	7
UPDRS Section 3	35	25	38	19

GDS = Geriatric Depression Scale; NART = National Adult Reading Test; UPDRS = Unified Parkinson's Disease Rating Scale.

**Table 2:** Questionnaire measures at baseline and follow-up.

Measure	Subscales	Participant	Time 1	Time 2	Time 3
PDEQ	Attention	P1	11	22	16
		P2	19	19	23
		P3	24	29	33
		P4	22	23	25

	Memory	P1	25	25	24
		P2	33	24	35
		P3	27	26	22
		P4	30	27	28

	Executive	P1	32	28	27
		P2	28	26	28
		P3	22	34	19
		P4	33	33	36

	Strategies	P1	12	14	10
		P2	17	19	22
		P3	4	13	11
		P4	20	18	20

PDEQ-carer		P1	74	88	93
		P2	100	109	99
		P3	96	77	91
		P4	116	101	105

PDQ-39 (0–100)	Total PDQ-39	P1	12.92	21.09	37.34
		P2	47.60	38.49	38.80
		P3	35.78	^*∗*^	^*∗*^
		P4	13.96	7.81	15.16

LARS (−36 ± 36)		P1	−28	−33	−33
		P2	−26	−21	−32
		P3	−32	−32	−30
		P4	−23	−26	−27

PDEQ = PD-Everyday Cognition Questionnaire; PDQ-39 = Parkinson's Disease Quality of Life Inventory-39; LARS = Lille Apathy Rating Scale. ^*∗*^Missing data.

**Table 3:** Open comments from questionnaire.

P1 Re tasks: “I was not clear what each was specifically trying to achieve ….”
P1: “The presence of the researchers each day meant that the exercises were undertaken and one can imagine it not being too difficult to find ways of avoiding doing it if one had the software on one's own PC.”
P1 Re changes in daily activity: “The exercises have given me pause for thought about my planning and subsequent implementation of larger projects such as planning a holiday… I have realised that I am not as sharp at planning and implementation … I am seeking to improve that. This realisation is a direct result of some of the exercises.”
P1 partner: “Since filling out the questionnaire I feel that I have become more aware of his behaviour and memory lapses,” “ we have both become more attuned to area where he is less able but he would need much more time and maybe training to improve.”
P2 Re changes in daily activity: “Try to concentrate more when doing tasks, and finish one task before starting another.”

**Table 4:** Overview of themes and codes (including occurrences).

Theme	Usefulness	Informational issues	Strategy use	Practical issues
Codes	Importance of addressing “thinking” issues (4)	Not enough information about task purpose (2)	Developed strategies to do the tasks (4)	Problems with technology interface (5)
	Didn't help with “thinking” beyond task (3)	More information about “thinking” that task represent (3)	Use feedback to develop strategies (1)	Problems with task instructions (2)
	Improved performance and “thinking” related to the task (2)	How do tasks relate to real life (1)	Need more information to inform strategies (2)	More options in terms of technology (1)
	Enjoyed doing the task (5)	More feedback on task performance (1)	More information on strategies that might be useful (1)	—
	Monitoring and awareness (3)	—		—

## Data Availability

Data generated in this study can be obtained from the corresponding author upon reasonable request and in line with the ethical approvals for this study.

## References

[1] Mamikonyan E., Moberg P. J., Siderowf A. (2008). Mild cognitive impairment is common in Parkinson’s disease patients with normal Mini-Mental State Examination (MMSE) scores. *Parkinsonism & Related Disorders*.

[2] Klepac N., Trkulja V., Relja M., Babić T. (2008). Is quality of life in non-demented Parkinson’s disease patients related to cognitive performance? A clinic-based cross-sectional study. *European Journal of Neurology*.

[3] Ziemssen T., Reichmann H. (2007). Non-motor dysfunction in Parkinson’s disease. *Parkinsonism & Related Disorders*.

[4] Leroi I., McDonald K., Pantula H., Harbishettar V. (2012). Cognitive impairment in Parkinson disease: impact on quality of life, disability, and caregiver burden. *Journal of Geriatric Psychiatry and Neurology*.

[5] Goetz C. G., Emre M., Dubois B. (2008). Parkinson’s disease dementia: definitions, guidelines, and research perspectives in diagnosis. *Annals of Neurology*.

[6] Buytenhuijs E. L., Berger H. J. C., Van Spaendonck K. P. M., Horstink M. W. I. M., Borm G. F., Cools A. R. (1994). Memory and learning strategies in patients with Parkinson’s disease. *Neuropsychologia*.

[7] Johnson A. M., Pollard C. C., Vernon P. A., Tomes J. L., Jog M. S. (2005). Memory perception and strategy use in Parkinson’s disease. *Parkinsonism & Related Disorders*.

[8] Poliakoff E., Smith-Spark J. H. (2008). Everyday cognitive failures and memory problems in Parkinson’s patients without dementia. *Brain and Cognition*.

[9] Hill N. L., McDermott C., Mogle J. (2017). Subjective cognitive impairment and quality of life: a systematic review. *International Psychogeriatrics*.

[10] Evans E. M., Llewellyn D. J., Matthews F. E., Woods R. T., Brayne C., Clare L. (2018). Social isolation, cognitive reserve, and cognition in healthy older people. *PLos One*.

[11] Emre M., Aarsland D., Albanese A. (2004). Rivastigmine for dementia associated with Parkinson’s disease. *New England Journal of Medicine*.

[12] Glizer D., MacDonald P. A. (2016). Cognitive Training in Parkinson’s disease: a review of studies from 2000 to 2014. *Parkinson’s Disease*.

[13] Sinforiani E., Banchieri L., Zucchella C., Pacchetti C., Sandrini G. (2004). Cognitive rehabilitation in Parkinson’s disease. *Archives of Gerontology and Geriatrics. Supplement*.

[14] Mohlman J., Chazin D., Georgescu B. (2011). Feasibility and acceptance of a nonpharmacological cognitive remediation intervention for patients with Parkinson disease. *Journal of Geriatric Psychiatry and Neurology*.

[15] Edwards J. D., Hauser R. A., O’Connor M. L., Valdes E. G., Zesiewicz T. A., Uc E. Y. (2013). Randomized trial of cognitive speed of processing training in Parkinson disease. *Neurology*.

[16] París A. P., Saleta H. G., de la Cruz Crespo Maraver M. (2011). Blind randomized controlled study of the efficacy of cognitive training in Parkinson’s disease. *Movement Disorders*.

[17] Sindhi A., Leroi I. (2013). Nonpharmacological therapies for cognitive enhancement in Parkinson’s disease: applying old interventions in a new setting?. *Neurodegenerative Disease Management*.

[18] Grewe P., Kohsik A., Flentge D. (2013). Learning real-life cognitive abilities in a novel 360°-virtual reality supermarket: a neuropsychological study of healthy participants and patients with epilepsy. *Journal of Neuroengineering and Rehabilitation*.

[19] Owen A. M., Hampshire A., Grahn J. A. (2010). Putting brain training to the test. *Nature*.

[20] Cerasa A., Gioia M. C., Salsone M. (2014). Neurofunctional correlates of attention rehabilitation in Parkinson’s disease: an explorative study. *Neurological Sciences*.

[21] McDonald K., Poliakoff E., Dick J. P., Kellett M. W., Crossman A. R. (2010). The development of a questionnaire to assess everyday multidomain cognitive functioning in people with Parkinson’s disease: the Parkinson’s disease everyday cognition questionnaire (PD-ECQ). *421 Movement Disorders*.

[22] Hoehn M. M., Yahr M. D. (1967). Parkinsonism: onset, progression, and mortality. *Neurology*.

[23] Moishi E., Dawson K., Mitchell J. (2006). The addenbrooke’s cognitive examination revised (ACE-R): a brief cognitive test battery for dementia screening. *International Journal of Geriatric Psychiatry*.

[24] Sheikh J. I., Yesavage J. A. (1986). In reply. *Psychiatric Services*.

[25] Nelson H. E. (1982). *National Adult Reading Test: Test Manual*.

[26] Raven J., Raven J. C., Court J. H. (1988). Manual for raven’s progressive matrices and vocabulary scales. *Section 2: The Coloured Progressive Matrices*.

[27] Lucza T., Ascherman Z., Kovács M. (2018). Comparing sensitivity and specificity of addenbrooke’s cognitive examination-I, III and mini-addenbrooke’s cognitive examination in Parkinson’s disease. *Behavioral Neurology*.

[28] Berankova D., Janousova E., Mrackova M. (2015). Addenbrooke’s cognitive examination and individual domain cut-off scores for discriminating between different cognitive subtypes of Parkinson’s disease. *Parkinson’s Disease*.

[29] Jenkinson C., Fitzpatrick R., Peto V., Greenhall R., Hyman N. (1997). The Parkinson’s Disease Questionnaire (PDQ-39): development and validation of a Parkinson’s disease summary index score. *Age and Ageing*.

[30] Sockeel P., Dujardin K., Devos D. (2006). The Lille apathy rating scale (LARS), a new instrument for detecting and quantifying apathy: validation in Parkinson’s disease. *Journal of Neurology, Neurosurgery & Psychiatry*.

[31] Deijen J. B., Stoffers D., Berendse H. W. (2006). Abnormal susceptibility to disctracters hinders perception in early stage Parkinson’s disease: a controlled study. *BMC Neurology*.

[32] Taylor M. M., Creelman C. D. (1967). PEST: efficient estimates on probability functions. *Journal of the Acoustical Society of America*.

[33] Jennings J. M., Jacoby L. L. (1997). Improving memory in older adults: training recollection. *Neuropsychological Rehabilitation*.

[34] Miles M. B., Huberman A. M. (1994). *Qualitative Data Analysis: An Expanded Sourcebook*.

[35] Olchik M. R., Farina J., Steibel N., Teixeira A. R., Yassuda M. S. (2013). Memory training (MT) in mild cognitive impairment (MCI) generates change in cognitive performance. *Archives of Gerontology and Geriatrics*.

[36] Calleo J., Burrows C., Levin H., Marsh L., Lai E., York M. K. (2012). Cognitive rehabilitation for executive dysfunction in parkinson’s disease: application and current directions. *Parkinson’s Disease*.

[37] Goebel S., Mehdorn H. M., Leplow B. (2010). Strategy instruction in Parkinson’s disease: influence on cognitive performance. *Neuropsychologia*.

[38] Clare L., Wilson B. A., Carter G., Roth I., Hodges J. R. (2004). Awareness in early-stage Alzheimer’s disease: relationship to outcome of cognitive rehabilitation. *Journal of Clinical and Experimental Neuropsychology*.

[39] Smith S. J., Souchay C., Moulin C. J. A. (2006). Awareness of prospective memory performance in Parkinson’s. *Neuropsychology*.

[40] Clare L., Wilson B. A., Carter G., Roth I., Hodges J. R. (2002). Assessing awareness in early-stage Alzheimer’s disease: development and piloting of the memory awareness rating scale. *Neuropsychological Rehabilitation*.

